# Tumor Lysis Syndrome Risk in Burkitt Lymphoma Versus Mantle Cell Lymphoma During Alkylating Therapy: A Propensity-Matched TriNetX Comparative Outcome Analysis

**DOI:** 10.7759/cureus.110145

**Published:** 2026-06-02

**Authors:** Anna Homeniuk, Gokul Karthikeyan, Anas Atrash

**Affiliations:** 1 Internal Medicine, University of Pittsburgh Medical Center, Harrisburg, USA; 2 College of Medicine, Drexel University, Philadelphia, USA

**Keywords:** alkylating agents, mantle cell lymphoma (mcl), primary burkitt lymphoma, propensity score matching (psm), real-world data, tumor lysis syndrome

## Abstract

Background

Tumor lysis syndrome (TLS) is a preventable oncologic emergency that can rapidly progress to acute kidney injury, malignant arrhythmias, seizures, and death. Because key prevention decisions (prophylaxis intensity, monitoring frequency, and inpatient versus outpatient initiation) are made at the start of therapy, clinically useful risk estimates must separate intrinsic disease risk from regimen-driven risk. However, cross-histology TLS comparisons are frequently confounded by systematic differences in chemotherapy composition and intensity. We therefore evaluated whether Burkitt lymphoma retains a higher TLS risk than mantle cell lymphoma (MCL) under a treatment-restricted framework anchored to shared alkylating exposure, with the goal of generating practical clinical insight for early TLS risk recognition, prevention, and monitoring in real-world practice, including urgent and emergency care settings.

Methods

We performed a retrospective cohort study using the TriNetX Global Collaborative Network, a federated real-world electronic health record research platform. Two cohorts were constructed (Burkitt lymphoma and MCL) and restricted to patients with exposure to cyclophosphamide or ifosfamide. Identical exclusions were applied to both cohorts for anthracyclines (doxorubicin, daunorubicin, epirubicin, idarubicin, and valrubicin), immune checkpoint inhibitors (nivolumab and pembrolizumab), and lymphoma coded as “in remission.” Outcomes were assessed beginning one day after cohort entry (index event).

The primary outcome was TLS (ICD-10-CM E88.3). Propensity score matching was performed 1:1. Time-to-event analyses used Kaplan-Meier methods with log-rank testing.

Results

After propensity score matching, 290 patients were included in each cohort (290 matched pairs). TLS occurred in 10.0% of the Burkitt cohort (29/290) compared with 5.5% of the mantle cell cohort (16/290), corresponding to an absolute risk difference of 4.5% (95% CI 0.1-8.8%; p = 0.044). On a relative scale, Burkitt lymphoma was associated with higher TLS risk, with a risk ratio of 1.813 (95% CI 1.006-3.264) and an odds ratio of 1.903 (95% CI 1.010-3.585), indicating a consistent direction and magnitude of effect across measures. Time-to-event analyses were concordant with the risk-based estimates: Kaplan-Meier analysis demonstrated shorter TLS-free survival in the Burkitt cohort (log-rank p = 0.034; HR 1.915, 95% CI 1.039-3.527). Median TLS-free survival was not reached in either cohort within available follow-up.

Conclusions

In this propensity-matched real-world cohort restricted to cyclophosphamide/ifosfamide exposure and excluding anthracyclines and immune checkpoint inhibitors, Burkitt lymphoma was associated with higher TLS incidence and shorter TLS-free survival compared with MCL. These findings support classifying Burkitt lymphoma as a higher-risk phenotype at treatment initiation and adopting more intensive TLS prevention and monitoring strategies (particularly proactive hydration and closer biochemical surveillance) with escalation of urate-lowering therapy as indicated, consistent with established TLS risk frameworks.

## Introduction

Tumor lysis syndrome (TLS) is an oncologic emergency caused by rapid tumor cell breakdown (spontaneous or treatment-related), leading to hyperuricemia, hyperkalemia, hyperphosphatemia, and hypocalcemia, with downstream risks, including acute kidney injury, malignant arrhythmias, seizures, and death [1,2]. TLS is also among the most actionable oncologic emergencies, as clinical outcomes are strongly influenced by anticipatory prevention strategies, such as hydration, urate-lowering therapy, and close biochemical surveillance instituted at or before treatment initiation [3-6]. Accordingly, early risk stratification directly informs the site of therapy initiation (inpatient vs. outpatient), laboratory monitoring frequency, and the selection and intensity of urate-lowering prophylaxis [3-5,7-9].

Contemporary TLS frameworks emphasize that risk reflects two interacting domains: intrinsic disease biology (tumor burden, proliferation rate, and chemosensitivity) and treatment-related factors (cytotoxic potency, pace of tumor kill, and supportive-care context) [3-5,10-12]. In real-world practice, however, clinicians often must make prophylaxis decisions at the start of therapy using incomplete information on tumor burden, lactate dehydrogenase (LDH), and baseline renal function, particularly during urgent admissions. As a result, early TLS precautions are frequently anchored on histology, which is consistently highlighted in risk-tiering schemes and institutional algorithms [3-5,10,11].

Burkitt lymphoma is a prototypical high-risk entity for TLS due to rapid proliferation and marked chemosensitivity, and it is consistently classified as high-risk in TLS stratification frameworks [1,3-5,12]. Mantle cell lymphoma (MCL), by contrast, demonstrates heterogeneous clinical behavior and biology. Although TLS is less frequently emphasized in MCL, it is recognized in the presence of additional risk modifiers such as bulky disease, high tumor burden, or high proliferative features [3-5,10-12].

A persistent practical challenge is that comparative TLS risk estimates across lymphoma subtypes are often difficult to interpret because regimen composition and intensity differ systematically by histology [3-5,10]. When treatment intensity varies substantially, observed TLS rates may reflect chemotherapy context rather than histology-driven biology. In clinical practice, however, physicians must rapidly determine the patient’s TLS risk in order to guide hydration strategy, urate-lowering therapy, monitoring frequency, and the need for inpatient treatment initiation [3-5,10-12].

To address this interpretability gap, we evaluated TLS risk in Burkitt lymphoma versus MCL using a treatment-restricted exposure framework anchored to shared alkylating therapy (cyclophosphamide or ifosfamide), while excluding exposures that may substantially alter TLS risk assessment or clinical context (anthracyclines and immune checkpoint inhibitors) [3-5]. We hypothesized that Burkitt lymphoma would retain a higher TLS risk even under partially harmonized chemotherapy exposure. The practical question motivating this study is therefore straightforward: when an alkylating agent is initiated, does histology still identify patients who warrant a lower threshold for intensive TLS prevention and monitoring?

## Materials and methods

We conducted a retrospective cohort study using TriNetX, a federated electronic health record research platform that supports real-world analyses across multiple healthcare organizations while maintaining site-level governance; data are represented as de-identified or pseudo-anonymized records depending on contributing site policy [6,7]. The analysis was performed using the TriNetX Global Collaborative Network, which included data from 150 healthcare organizations. Patients were identified using diagnostic and medication codes recorded within the TriNetX electronic health record system.

Two cohorts were constructed using the TriNetX Compare Outcomes workflow: patients with Burkitt lymphoma and patients with MCL. Cohort definitions were based on ICD-10-CM diagnostic codes for Burkitt lymphoma and MCL, including site-specific lymphoma codes. To reduce treatment heterogeneity and improve the interpretability of cross-histology TLS comparisons, both cohorts were restricted to patients with documented exposure to at least one alkylating agent, defined as the presence of a medication record for cyclophosphamide or ifosfamide in the TriNetX electronic health record system. The exposure definition was binary/documentation-based rather than dose-based; cumulative dose, number of cycles, treatment duration, and regimen-level intensity were not available in the Compare Outcomes output and were not used to define exposure.

To preserve temporal sequencing, the first recorded instance of cyclophosphamide or ifosfamide was required to occur on or after any recorded lymphoma diagnosis. Thus, patients entered the treatment-restricted cohort only if alkylating therapy was documented after the lymphoma diagnosis. The analysis did not require a minimum number of doses and did not impose a fixed exposure window beyond this temporal relationship, because the objective was to identify TLS risk among patients with documented initiation or receipt of shared alkylating therapy rather than to evaluate dose-response effects.

To further reduce confounding from therapies that may represent substantially different treatment contexts and influence TLS risk assessment at treatment initiation, identical exclusions were applied to both cohorts. Patients were excluded if they had medication records for selected anthracyclines or immune checkpoint inhibitors, including doxorubicin, daunorubicin, epirubicin, nivolumab, or pembrolizumab, or if they had lymphoma coded as “in remission.” These exclusions were applied symmetrically to both histology cohorts to reduce regimen-driven and disease-status heterogeneity.

The initial analytic cohort identified 421 patients with Burkitt lymphoma and 649 patients with MCL who met the defined inclusion criteria.

The index event was defined using the cohort criteria within the TriNetX platform, with the index date corresponding to the first qualifying event meeting the cohort definition. Outcomes were assessed beginning one day after cohort entry to focus on post-index TLS events and reduce the likelihood of including pre-existing TLS at baseline. No predefined end date was specified for the time window; therefore, outcomes occurring after the index event were included throughout the available follow-up period within the platform. In time-to-event analyses, patients were censored according to available follow-up within the TriNetX platform, which reflects the last available recorded fact or encounter in the EHR. The TriNetX platform restricts index events to those occurring within the previous 20 years.

The primary outcome was tumor lysis syndrome, defined using ICD-10-CM code E88.3 [1-5].

Because baseline demographic characteristics differed between cohorts prior to adjustment, 1:1 propensity score matching was performed using the TriNetX built-in matching workflow. The propensity score model included demographic characteristics available in the platform and selected for matching in this analysis: age, age at index, sex, and race categories. After matching, 290 patients remained in each cohort. Balance was assessed using standardized differences, with values <0.1 considered acceptable. After matching, all included demographic variables had standardized differences <0.1, supporting improved comparability between cohorts for outcome estimation. Disease stage, tumor burden, lactate dehydrogenase, baseline renal function, uric acid level, phosphate level, potassium level, prior lines of therapy, previous lymphoma-directed treatment, and prophylaxis use were not included in the propensity score model because these variables were not available or not consistently extractable in the TriNetX Compare Outcomes output used for this analysis.

Statistical analyses were performed within the TriNetX platform. Effect estimates included event proportions, absolute risk difference with 95% confidence intervals, risk ratios, odds ratios, and corresponding p-values. Time-to-event analyses were performed using Kaplan-Meier methods with log-rank testing and hazard ratio reporting. Additional analyses included evaluation of the number of outcome instances within the follow-up period. A two-sided p-value <0.05 was considered statistically significant.

Because the TriNetX platform provides access only to de-identified or pseudo-anonymized data, this study was considered exempt from institutional review board oversight [6,7].

## Results

After propensity score matching, 290 patients were included in each cohort (290 matched pairs). Before matching, the Burkitt cohort was younger and differed in sex and race distribution compared with the MCL cohort (Table 1).

**Table 1:** Baseline demographics before propensity score matching

Characteristic	Burkitt (N = 421)	Mantle cell (N = 649)	P-value	Std. diff.
Age, years				
Current age, mean ± SD	59.9 ± 21.5	71.3 ± 11.6	<0.001	0.659
Age at index, mean ± SD	51.6 ± 21.4	64.9 ± 12.1	<0.001	0.768
Race, n (%)				
White	257 (61.0%)	486 (74.9%)	<0.001	0.255
Black/African American	38 (9.0%)	23 (3.5%)	<0.001	0.238
Asian	18 (4.3%)	26 (4.0%)	0.739	0.021
Other race	22 (5.2%)	27 (4.2%)	0.347	0.059
Unknown race	86 (20.4%)	87 (13.4%)	0.083	0.109
Sex, n (%)				
Female	132 (31.4%)	152 (23.4%)	0.002	0.193
Male	289 (68.6%)	497 (76.6%)	0.004	0.129

After matching, demographic variables showed improved balance with small standardized differences, supporting comparability of the matched cohorts for outcome estimation (Table 2). 

**Table 2:** Baseline demographics after propensity score matching (290 matched patients per cohort)

Characteristic	Burkitt (N = 290)	Mantle cell (N = 290)	P-value	Std. diff.
Age, years				
Current age, mean ± SD	68.4 ± 16.2	67.9 ± 12.7	0.663	0.036
Age at index, mean ± SD	60.7 ± 15.6	60.3 ± 13.1	0.767	0.025
Race, n (%)				
White	212 (73.1%)	207 (71.4%)	0.643	0.039
Black/African American	17 (5.9%)	19 (6.6%)	0.731	0.029
Asian	14 (4.8%)	12 (4.1%)	0.688	0.033
Other race	10 (3.4%)	12 (4.1%)	0.664	0.036
Unknown race	37 (12.8%)	40 (13.8%)	0.808	0.02
Sex, n (%)				
Female	83 (28.6%)	77 (26.6%)	0.513	0.054
Male	207 (71.4%)	213 (73.4%)	0.583	0.046

All standardized differences after matching were <0.1, indicating good covariate balance between cohorts.

In the matched analysis, TLS occurred in 10.0% of patients in the Burkitt cohort (29/290) compared with 5.5% in the MCL cohort (16/290), corresponding to an absolute risk difference of 4.5% (95% CI 0.1%-8.8%; p = 0.044). The association was consistent across relative effect measures, with a risk ratio of 1.813 (95% CI 1.006-3.264) and an odds ratio of 1.903 (95% CI 1.010-3.585).

**Figure 1:**
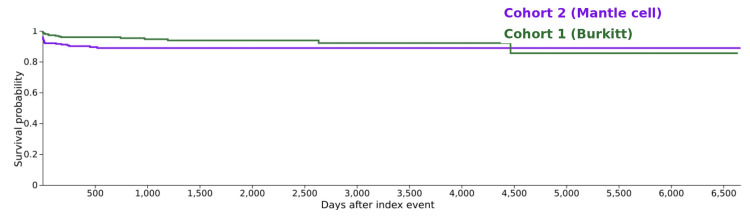
Kaplan–Meier tumor lysis syndrome (TLS)-free survival during follow-up in Burkitt lymphoma versus mantle cell lymphoma.

Time-to-event analysis yielded concordant results. Kaplan-Meier estimates demonstrated significantly shorter TLS-free survival in the Burkitt cohort compared with the MCL cohort (log-rank p = 0.034), with a hazard ratio of 1.915 (95% CI 1.039-3.527) (Figure 1).

Median TLS-free survival was not reached in either cohort during the available follow-up period.

Among patients who developed TLS, the mean number of coded TLS instances did not differ significantly between cohorts. This suggests that the primary difference between groups was the probability of developing TLS rather than the frequency of repeated TLS-coded encounters among affected patients.

## Discussion

**Figure 2:**
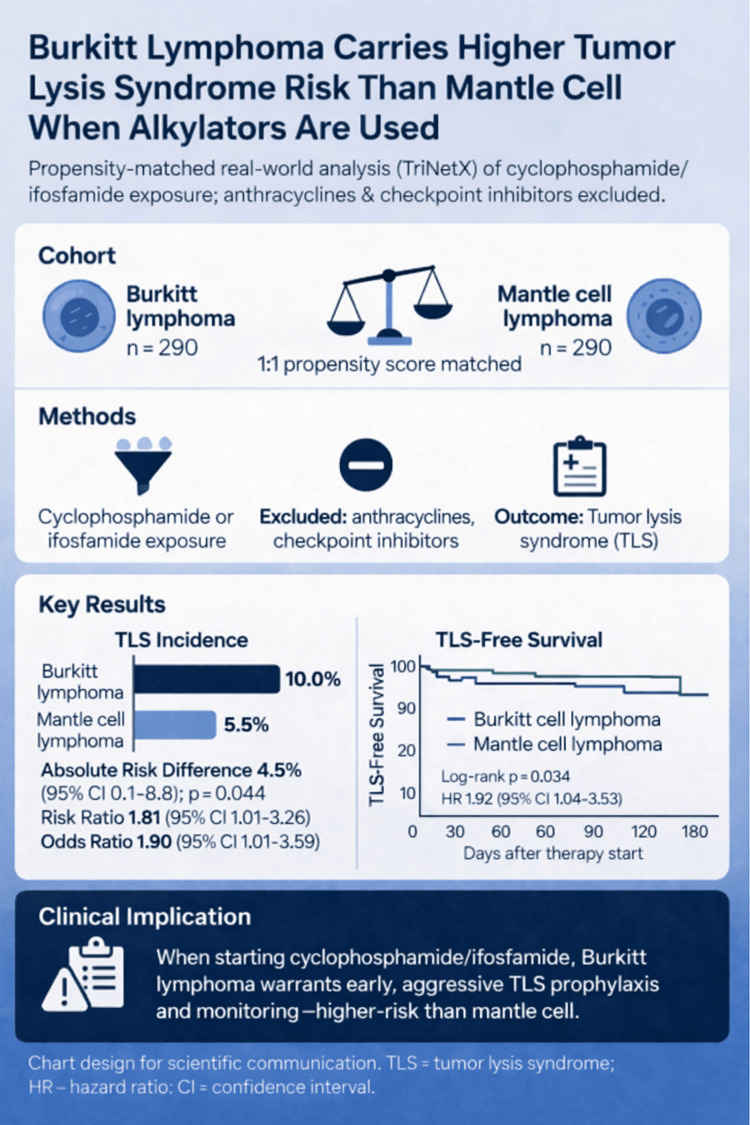
Graphical abstract. Tumor lysis syndrome risk in Burkitt lymphoma versus mantle cell lymphoma during alkylating therapy. Image created using Canva (web-based graphic design platform; Canva Pty Ltd., Australia), author: Anna Homeniuk.

In this propensity-matched real-world cohort restricted to cyclophosphamide/ifosfamide exposure and applying symmetric exclusions for anthracyclines and immune checkpoint inhibitors, Burkitt lymphoma was associated with a significantly higher incidence of coded TLS and shorter TLS-free survival than MCL. The excess risk was consistent on both absolute and relative scales and supported by concordant Kaplan-Meier analysis. This direction of effect is biologically plausible and aligns with established TLS frameworks that emphasize the interaction of intrinsic tumor biology, particularly proliferation rate and chemosensitivity, with treatment exposure [1,3-5,12] (Figure 2). 

The principal contribution of this study is improved clinical interpretability. In many real-world comparisons, histology-associated differences in treatment intensity make TLS risk estimates difficult to apply at therapy initiation, when clinicians must determine monitoring location and prophylaxis intensity [3-5,10-12]. By anchoring both cohorts to shared alkylating exposure and applying symmetric exclusions, our design reduces a major source of regimen-driven confounding and more directly evaluates whether histology remains a practical discriminator of TLS risk within a partially harmonized treatment context. This approach aligns with how TLS risk is operationalized in clinical algorithms and consensus guidance, which link risk stratification to hydration strategies, urate-lowering therapy selection, and laboratory monitoring frequency [3-5,13].

These findings are clinically relevant because TLS prevention is inherently front-loaded. Evidence-based recommendations and consensus guidance consistently emphasize that prevention - through hydration, appropriate urate-lowering therapy, and close biochemical monitoring - is the most effective strategy for reducing TLS-related morbidity. In settings of urgent treatment initiation, where complete risk modifiers such as tumor burden, LDH, and renal function may not yet be fully available, a treatment-restricted comparative estimate supports a conservative clinical approach. Specifically, Burkitt lymphoma may warrant a lower threshold for heightened TLS risk recognition, early preventive strategies, and closer biochemical monitoring than MCL when initiating alkylating therapy, consistent with guideline-based risk frameworks.

The “instances” analysis provides additional context. Among patients who developed TLS, the mean number of coded TLS instances did not differ significantly between cohorts. This suggests that the primary difference observed in this study was the probability of TLS occurrence rather than the frequency of repeated TLS-coded encounters among affected patients. From a prevention perspective, these findings support prioritizing early identification of high-risk patients and implementing prophylactic strategies to reduce the number of patients who develop TLS initially [3-5,10-12].

Several limitations warrant consideration. First, TLS was defined using ICD-10-CM coding (E88.3), and code-based ascertainment may under-capture laboratory TLS, misclassify event timing, and vary across institutions [1-5]. Second, although propensity matching improved balance across available baseline characteristics, residual confounding may persist for variables not captured or not included in the matching process, including tumor burden, LDH, baseline renal function, baseline uric acid and electrolyte values, prophylaxis use, prior lines of therapy, previous lymphoma-directed treatment, and treatment intensity beyond alkylator exposure. These variables are clinically important because renal dysfunction, high tumor burden, elevated LDH, prior treatment context, and the use or absence of TLS prophylaxis may substantially influence observed TLS risk. In addition, alkylating exposure was defined by documentation of cyclophosphamide or ifosfamide receipt rather than cumulative dose, number of cycles, dose intensity, or complete regimen structure, which may introduce residual treatment-related confounding. Third, TriNetX is a federated platform; contributing sites differ in documentation practices and follow-up patterns, and time-to-event estimates reflect EHR encounter structures rather than protocolized surveillance [6,7,9]. Therefore, the findings should be interpreted as a real-world association rather than evidence that histology alone independently determines TLS risk, and clinical application should be individualized based on renal function, tumor burden, LDH, baseline laboratory values, prior treatment, and prophylaxis strategy. Future studies incorporating baseline renal function, laboratory TLS risk markers, prophylaxis strategy, prior treatment exposure, and regimen-level treatment details are needed to provide a more comprehensive assessment of TLS risk across lymphoma subtypes. Nevertheless, the consistency across absolute, relative, and time-to-event estimates supports the observed association within the constraints of real-world data.

## Conclusions

In a propensity-matched federated EHR cohort restricted to cyclophosphamide/ifosfamide exposure and excluding anthracyclines and immune checkpoint inhibitors, Burkitt lymphoma was associated with significantly higher coded TLS incidence and shorter TLS-free survival compared with mantle cell lymphoma. These findings support a lower threshold for intensified TLS prevention and biochemical monitoring at treatment initiation in Burkitt lymphoma relative to MCL, consistent with established TLS risk frameworks and contemporary practice guidance. 

When initiating cyclophosphamide or ifosfamide, Burkitt lymphoma should be approached as having a higher TLS risk than mantle cell lymphoma, even after matching, supporting early proactive prevention (hydration, monitoring, and urate-lowering escalation as indicated) rather than reactive management. 
